# A surface plasmon resonance nanostructure containing graphene and BaTiO_3_ layers for sensitive defection of organic compounds

**DOI:** 10.1098/rsos.230282

**Published:** 2023-06-21

**Authors:** Sofyan A. Taya, Malek G. Daher, Abdulkarem H. M. Almawgani, Ayman Taher Hindi, Ilhami Colak

**Affiliations:** ^1^ Physics Department, Islamic University of Gaza, P.O. Box 108, Gaza, Palestine; ^2^ Electrical Engineering Department, College of Engineering, Najran University, Najran, Kingdom of Saudi Arabia; ^3^ Department of Electrical and Electronics Engineering, Nisantasi University, Istanbul, Turkey

**Keywords:** surface plasmon resonance, organic compounds, BaTio_3_, graphene, chemical sensor

## Abstract

Organic compound-based sensors are used in a variety of significant fields, including medical research, azeotropic calibration, vegetable oil extraction, the shoe industry and geothermal power plants. Here, a high-performance, two-dimensional material-based organic compound sensor has been proposed using a surface plasmon resonance (SPR) nanostructure consisting of a BK7 glass prism, Ag, BaTiO_3_, Ag, graphene and sensing layer. The reflectivity curves of the SPR device have been investigated when the sensing media are Pentane, n-Hexane, n-Heptane and n-Octane. The thickness of the BaTiO_3_ layer and the number of graphene sheets have been optimized to maximize the sensitivity. The highest sensitivity attained is 220.83 deg/RIU for n-Octane with 45 nm silver/10 nm BaTiO_3_/8 nm silver and four layers of graphene. We believe that the SPR-based sensors are simple and can replace the spectrometry, chromatography and electrochemical based sensors. The proposed design is extremely effective for diverse applications in biological, industrial and chemical detection because of its simple structure and great performance.

## 1. Introduction

Recently, optical technology has been extensively employed in the field of interferometry [1], ellipsometry [2], spectroscopy [3], etc. Surface plasmon resonance (SPR) sensors are mostly used in applications for chemical and biological detection [4]. There has been a lot of interest in the SPR sensor because of its superior real-time sensing capability and label-free characteristics [5]. SPR has many uses, including environmental protection, food safety, gas detection, etc. Kretshmann configuration [6] is usually employed in SPR-based devices. In this configuration, incident light travels through a prism of glass, reflects off the backside of the sensor chip, and finally is received by a detector. Metal electrons can absorb part of the incoming light at a particular angle of incidence. The angle at which absorption takes place is called the resonance angle (RA), which causes the electrons to resonate [7]. As minor modifications in the refractive index (RI) result in a significant shift in the RA, SPR sensors are commonly used in molecular and biological interactions [8]. With noble two-dimensional nanomaterials, several SPR sensor designs have been investigated. On the dielectric medium, reflection and SPR angles have been obtained using metals (Au, Ag, etc.). The SPR sensor has been examined using angular interrogation for a fixed wavelength [9]. Due to their energy-efficient, light weight and great conducting properties, two-dimensional materials have been used widely in SPR sensors [10]. Previous studies have claimed that using two-dimensional nanomaterials like Perovskite materials and transition metal dichalcogenides (TMDEs) [11], graphene [12–15], Franckeite nanosheets [16], Black phosphorus [17] and Tin selenide [18] has dramatically improved the SPR sensor performance. It was shown that adding a graphene layer to the SPR sensor structure can improve the tuning range and sensitivity [19] as a protection layer to the plasmonic metal from oxidation and this can lead to more stability of the performance. Typically, two-dimensional/TMD heterostructures or Perovskite materials are employed in metal prism-based biosensors [20–25]. The two-dimensional nanomaterial BaTiO_3_ is a desirable option for thin-film electro-optic switches because it has substantial electro-optical coefficients [26]. It is an inorganic compound that appears white as a powder and it is transparent when prepared as large crystals. BaTiO_3_ nano-films have been made using bottom-up and top-down manufacturing techniques over the past 20 years [27].

One of the most essential chemical substances is an organic compound (OCD), which is widely employed in our daily lives in many fields including chemical industry, food industry, disease diagnosis, biotechnology, etc. Although OCDs are useful for industry, some of them are so harmful when released directly into the environment. OCDs can also be used in the chemical industry and their chemical waste can pose a serious threat to human health as well as being a source of gaseous environmental pollution. OCD pollution causes negative side effects and has become a source of serious human illnesses. Therefore, it is essential to develop techniques that enable prompt detection of organic chemicals in order to stop the spread of their harmful effects. Several traditional approaches are currently available for the detection of organic molecules such as chromatography-mass spectrometry, solvent-response material, quartz crystal microbalance, electronic nose and fluorescence probes [28]. These techniques require complicated device setup and suffer from other technological drawbacks including difficult handling, high cost and time-consuming operation.

The aim of the current work is to propose a simple and accurate SPR-based sensor for the detection of four OCDs: n-Octane, n-Heptane, n-Hexane and Pentane. The OCD pentane has the chemical formula C_5_H_12_, which designates it as an alkane with five carbon atoms. The blowing agent created from pentane is used to make a foam called polystyrene. Insulation materials for heating pipes and freezers are made from polystyrene. It is a colourless liquid and it smells faintly of gasoline. It is utilized in the production of low-temperature thermometers, polymers and other compounds. It is toxic to aquatic living and can cause long-term effects in the aquatic environment. A deficiency of oxygen can be caused by high concentrations of pentane in the air which can lead to the risk of unconsciousness or death. Crude oil is used to create the OCD n-Hexane. Pure n-Hexane has an annoying odour. It is a flammable OCD and its vapour is explosive. The main application for solvents containing n-Hexane is the extraction of vegetable oils from crops such as soya beans. N-heptane is a colourless liquid that smells like petroleum. It is insoluble in water and less dense than water. Its vapours are heavier than air. It is employed in petroleum refining procedures and as an industrial solvent. The Hazardous Substance List includes n-Heptane because Occupational Safety and Health Administration in the USA regulates it. It is also flammable. N-octane is an odour of gasoline and a colourless liquid. It is insoluble in water and less dense than water. Its vapour is irritating. Concentrated vapour inhalation can result in pulmonary edema, depression and respiratory tract irritation. Liquid can irritate the eyes and, after continuous contact, the skin, leading to inflammation and skin cracking. Mouth and stomach irritation can result from ingestion. Aspiration results in acute lung irritation.

In this paper, we propose an enhanced sensitivity SPR-based sensor for the detection of four OCDs. These compounds are n-Octane, n-Heptane, n-Hexane and Pentane. The structure is based on BaTiO_3_ and graphene layers.

## 2. Theoretical model

**Figure 1.**
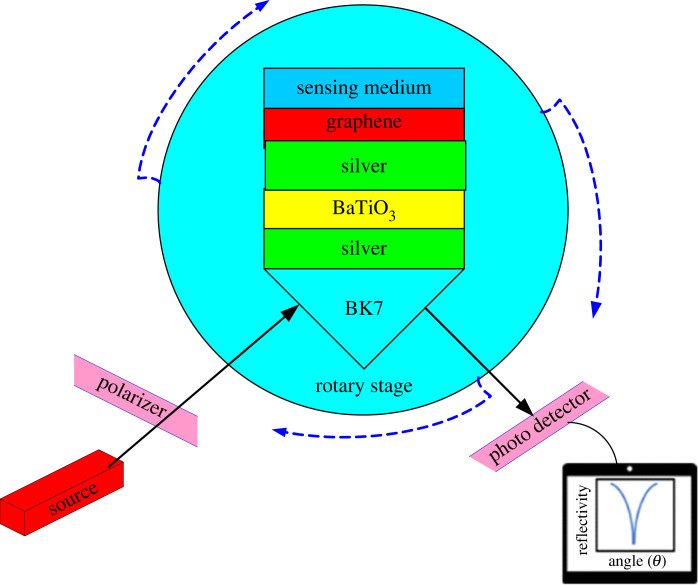
An SPR chemical sensor based on Ag- BaTiO_3_-Ag-graphene layers.

A BK7 glass prism (*n*_p_ is the RI) is employed to couple an incident laser beam of wavelength (*λ*) of 632.8 nm into the SPR-based device. BK7 is an excellent option for windows, substrates or wafers since it is a pure optical borosilicate-crown glass. It is supplied for industrial, medical, and high precision lower power laser applications primarily for the visible spectrum and near IR, with specific coatings used for enhanced transmission. The proposed detector has the following structure: BK7 glass prism, Ag, BaTiO_3_, Ag, graphene and sensing layer (figure 1). The thicknesses of Ag (first layer), BaTiO_3_, Ag (second layer) and graphene are symbolized as *d*_1,_ *d*_2,_ *d*_3_ and *d*_4_ and the RIs are designated as *n*_1_, *n*_2_ *n*_3_ and *n*_4_, respectively. The thickness of the sensing medium layer is very large compared to those thicknesses so that it is considered to have an infinite thickness.

The RI of BK7 glass prism is given by [29]

2.1 $$n(\lambda )=\sqrt{1+\frac{s_{1}\;\lambda^{2}}{\lambda^{2}-g_{1}}+\frac{s_{2}\;\lambda^{2}}{\lambda^{2}-g_{2}}+\frac{s_{3}\;\lambda^{2}}{\lambda^{2}-g_{3}}},$$

where *s*_1_ = 1.03961212, *s*_2_ = 1.01046945, *s*_3_ = 0.231792344, *g*_1_ = 0.00600069867, *g*_2_ = 103.560653 and *g*_3_ = 0.0200179144. The wavelength is inserted in equation (2.1) in μm. The RI of the Ag layer depends on *λ* according to [30]

2.2 $$n_{\mathrm{Ag}}(\lambda )={\left(1-\frac{\lambda_{c}\;\lambda^{2}}{\lambda_{p}^{2}(\lambda_{c}+i\lambda \;)}\right)}^{1/2},$$

where *λ*_c_ = 17.614 µm and *λ*_p_ = 0.14541 µm are the silver collision and plasma wavelengths. The RI of BaTiO_3_ is 2.4043. The graphene RI as a function of *λ* is given by [31]

2.3 $$n_{G}(\lambda )=3+\lambda \frac{C}{3}i,\;C=5.446\;\mu m^{-1}.$$

The wavelength is inserted into equations (2.2) and (2.3) in μm.

Only transverse magnetic (TM) polarization in naturally occurring materials allows the SPR phenomenon. The proposed structure was examined utilizing the angular modulation approach. Analysis of the reflectivity of the incident light has been conducted using the transfer matrix (TRM) approach and Fresnel multilayer reflection theory. The propagation constants of the surface plasmon and the p-polarized incident wave must be equal for the surface plasmon to be excited. This condition can be expressed mathematically as [6]

2.4 $$\frac{\omega }{c}\sqrt{\varepsilon_{p}}\;\sin\theta_{\mathrm{in}}=\frac{\omega }{c}\sqrt{\frac{\varepsilon_{\mathrm{Ag}}\varepsilon_{d}}{\varepsilon_{\mathrm{Ag}}+\varepsilon_{d}}},$$

where *ε*_p_, *ω*, *ε*_Ag_, *θ*_in_ and *ε*_d_ are the permittivity of the prism, incident light frequency, metal permittivity, incident angle and the dielectric permittivity.

The tangential *E*- and *H*-field components at the first and last boundaries are related to each other by [28]

2.5 $$\left[\begin{array}{c} E_{1}\\ H_{1} \end{array}\right]=E_{T}\left[\begin{array}{c} E_{N-1}\\ H_{N-1} \end{array}\right],$$

where *E*_T_ is the TRM. The TRM (*E*_j_) of the *j*th layer is given as [28]

2.6 $$E_{j}=\left[\begin{array}{cc} \cos(X_{j}) & -\frac{i\;\sin(X_{j})}{U_{j}}\\ -iU_{j}\sin(X_{j}) & \cos(X_{j}) \end{array}\right],$$

*X_j_* is the phase shift which is given by

2.7 $$X_{j}=\frac{2\pi }{\lambda }d_{j}{\left(\varepsilon_{j}-{\left(n_{1}\sin\theta_{1}\right)}^{2}\right)}^{0.5},$$

where *n*_1_ and *θ*_1_ are the RI and the incident angle of the prism. *U_j_* is given for TM polarization as

2.8 $$U_{j}=\frac{{\left(\varepsilon_{j}-{\left(n_{1}\sin\theta_{1}\right)}^{2}\right)}^{0.5}}{\varepsilon_{j}}.$$

The system TRM (*E_T_*) of the structure

2.9 $$E_{T}=E_{1}\;E_{2}\;E_{3}\;E_{4}=\left[\begin{array}{cc} e_{11} & e_{12}\\ e_{21} & e_{22} \end{array}\right],$$

where *E*_1_, *E*_2_, *E*_3_ and *E*_4_ are the TRMs of Ag, BaTiO_3_, Ag and graphene.

The reflection coefficient (*r*), in terms of *e_ij_*, can be written as

2.10 $$r=\frac{\left(e_{11}+e_{12}U_{s})U_{p}-(e_{21}+e_{22}U_{s}\right)}{\left(e_{11}+e_{12}U_{s})U_{p}+(e_{21}+e_{22}U_{s}\right)},$$

where *U*_s_ and *U*_p_ are obtained from equation (2.8) for the sensing medium and prism.

The reflectance (*R*) of the nanostructure can be written as

2.11 $$R=r.r^{*}=|r{|}^{2}.$$

It is common to calculate the sensitivity (*S*), detection accuracy (DA), full width at half maximum (FWHM) and figure of merit (FoM) to evaluate the SPR sensor performance. The RA (*θ*_res_) shifts as a result of the sensing medium (SM) RI changing. The sensitivity of an SPR sensor is determined by both (Δ*n*) and RA shift (Δ*θ*_res_) as

2.12 $$S=\frac{\;\Delta \theta_{\mathrm{res}}}{\Delta n}.$$

The FWHM can also be determined using the reflectance curve. It is calculated as

2.13 $$\mathrm{FWHM}=\;\theta_{2}-\theta_{1}$$

where *θ*_1_ and *θ*_2_ are the RAs at 50% reflectance.

DA is given by

2.14 $$\mathrm{DA}=\frac{1}{\mathrm{FWHM}}.$$

The FoM is the product of *S* and DA of the sensor as

2.15 FoM=S×DA.

## 3. Results and discussion

**Figure 2.**
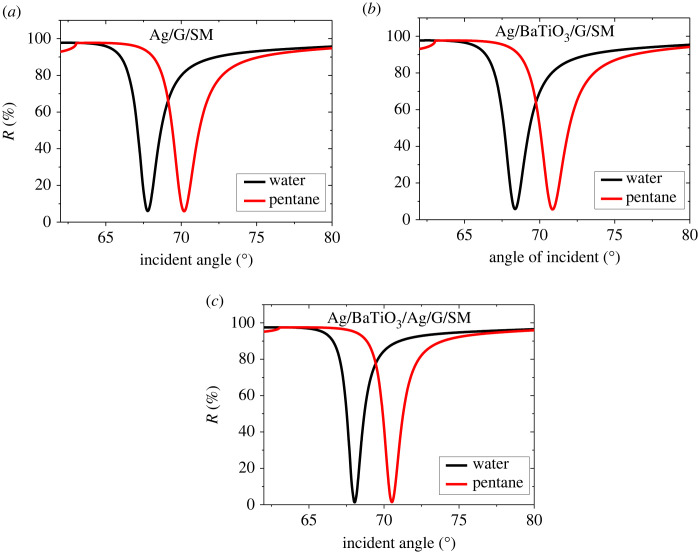
RECs of the SPR sensors for different assemblies (*a*) Ag/G/SM (*b*) Ag/BaTiO_3_/G/SM and (*c*) Ag/BaTiO_3_/Ag/G/SM.

For the detection of the above-mentioned OCDs, an optical chemical sensor based on SPR structure is investigated. A transverse magnetic (TM) beam is incident on the SPR structure as shown in figure 1. The calculations of the RIs are conducted at 632.8 nm wavelength. The thicknesses of BaTiO_3_ and graphene (G) layers are initially chosen as *d*_2_ = *P* × 1.0 nm and *d*_4_ = *M* × 0.34 nm where *P* = 1 and *M* = 1. The thicknesses of the Ag layers have been taken as *d*_1_ = 45 nm and *d*_3_ = 8 nm. The RIs of the OCDs are 1.39, 1.38, 1.37 and 1.35 for n-Octane, n-Heptane, n-Hexane and Pentane, respectively [28]. Figure 2 displays the reflectance curves (RECs) of three different sensor configurations for two analytes (water and pentane) where the RI of water is 1.33. The three panels demonstrate that the resonance dip shifts to higher RAs as the RI of the analyte increases. Figure 2*a* displays the RECs of an SPR sensor having the assembly prism/Ag/G/ SM (assembly 1). An RA of 67.78 deg is attained for an SM of RI of 1.33. A minute rise in the RI of the analyte (Δ*n* = 0.02) shifts the RA to 70.19 deg. Using equation (2.12), the sensitivity (S in deg/RIU) is found for structure 1 as 120.5. FWHM, DA and FoM are attained for structure 1 from the REC (figure 2*a*) as 1.8 deg, 0.555 deg^−1^ and 66.94 RIU^−1^, respectively. After inserting BaTiO_3_ in structure 1 sensor, the assembly becomes prism/ Ag/ BaTiO_3_/ G/ SM (assembly 2). Figure 2*b* plots the REC of assembly 2. In this case, the sensitivity realized is 123 deg/RIU. FWHM, DA and FoM attained for assembly 2 are 1.86 deg, 0.537 deg^−1^ and 66.12 RIU^−1^. The sensitivity improvement due to introducing a layer of BaTiO_3_ between the graphene and metal layers is 2.07%. When a silver layer of thickness 8 nm is introduced between the BaTiO_3_ layer and the monolayer of graphene, the structure becomes prism/ Ag/ BaTiO_3_ /Ag /G / SM (assembly 3) and the REC has the configuration shown in figure 2*c*. A higher sensitivity of 125 deg/RIU is achieved with an improvement of 3.73% and 1.62% over structure 1 and structure 2. The higher sensitivity of assembly 3 is accredited to using a high RI medium at the analyte interface. Inserting a BaTiO_3_ layer leads to the enhancement of the field intensity at the analyte interface. The field intensity at the material interface should be as high as feasible to effectively identify a substance. FWHM, DA and FoM are obtained from the REC (figure 2*c*) as 1.22 deg, 0.819 deg^−1^ and 102.45 RIU^−1^. Performances for all assemblies are reported in table 1.

**Table 1.** The assemblies and their performance parameters (FWHM, DA, S and FoM).

assembly no.	the used assembly	FWHM (deg)	DA (deg^−1^)	*S* (deg/RIU)	FoM (RIU^−1^)
1	Ag/G/SM	1.8	0.555	120.5	66.94
2	Ag/BaTiO_3_/G/SM	1.86	0.537	123	66.12
3	Ag/BaTiO_3_/Ag/G/SM	1.22	0.819	125	102.45

**Figure 3.**
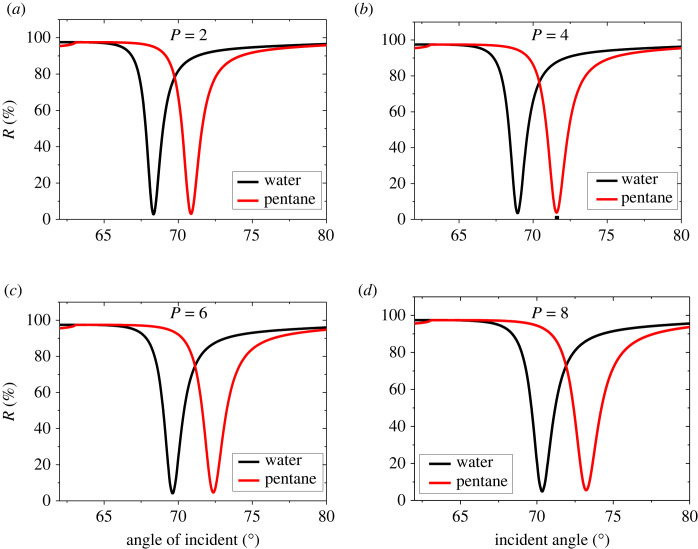
RECs of the device Prism/Ag/BaTiO_3_/Ag/G/SM at different thicknesses of BaTiO_3_. (*a*) *P* = 2 (*b*) *P* = 4 (*c*) *P* = 6 and (*d*) *P* = 8. The SM is water and Pentane.

**Figure 4.**
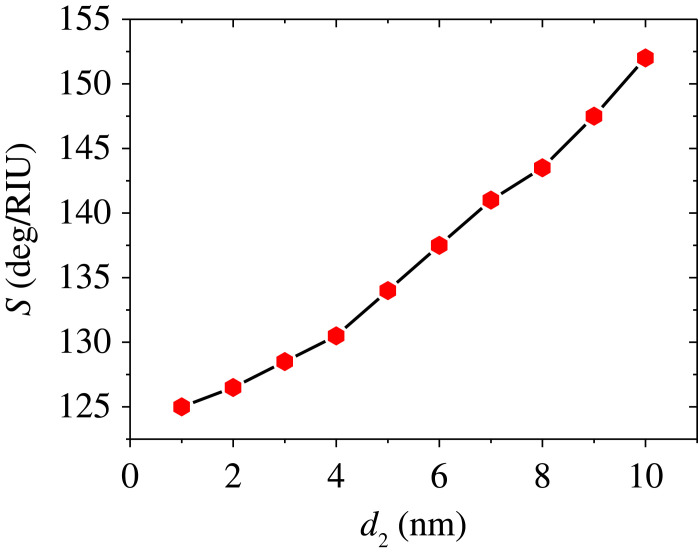
Sensitivity of the device Prism/Ag/BaTiO_3_/Ag/G/SM versus the thickness of BaTiO_3_ at *d*_1_ = 45 nm, *d*_3_ = 8 nm and *d*_4_ = *M* × 0.34 nm (*M* = 1).

A suitable selection of the system layer thicknesses is necessary for maximizing the SPR sensor sensitivity. Therefore, in the following, we examine various choices for the structure layer thicknesses. Three significant thicknesses play a crucial role in the device performance. We start systematically investigating the BaTiO_3_ thickness effect on the sensitivity for thicknesses of Ag of 45 nm (first layer) and 8 nm (second layer) and a mono graphene layer. The assembly is now characterized by the following parameters: *d*_1_ = 45 nm, *d*_3_ = 8 nm and *d*_G_ = *M* × 0.34 nm where *M* = 1 and SM is selected as water and pentane. The thickness of the BaTiO_3_ layer is assumed to be *d*_2_ = P × 1.0 nm where *P* is varied from 1 to 10 by a step of 1. The RECs are illustrated in figure 3 for the thicknesses of BaTiO_3_ of *P* = 2 (figure 3*a*), *P* = 4 (figure 3*b*), *P* = 6 (figure 3*c*) and *P* = 8 (figure 3*d*). The increase in the thickness of BaTiO_3_ leads to an increase in the REA as the dips moves to higher angles as can be seen in figure 3. However, it is seen that the sensitivity enhances from 125 to 152 deg/RIU as the thickness of BaTiO_3_ changes from *P* = 1 to *P* = 10. This enhancement is due to the increase in the REA as the thickness of BaTiO_3_ increases. The resonant angle position, angular shift, and sensitivity corresponding to the considered thicknesses of BaTiO_3_ layer are presented in table 2. For values of *P* > 10, no dip can be observed in the RECs. Figure 4 represents the variation of the sensitivity for different thicknesses of the BaTiO_3_ layer. From this figure, it is found that the sensitivity is enhanced by increasing the BaTiO_3_ layer thickness. The sensitivity becomes maximum at *P* = 10 (*d*_2_ = 10 nm). Therefore, the optimized BaTiO_3_ layer thickness occurs when *P* = 10 (*d*_2_ = 10 nm) at which the proposed SPR sensor shows the best performance. Thus, it is noted that *P* = 10 will be used in the following steps.

**Table 2.** Variation of the sensitivity of the device Prism/Ag/BaTiO_3_/Ag/G/SM at different thicknesses of BaTiO_3_ at *d*_1_ = 45 nm, *d*_3_ = 8 nm and *d*_4_ = *M* × 0.34 nm (*M* = 1).

thickness of BaTiO_3_ (nm)	position of resonant dip for water (deg)	position of resonant dip for pentane (deg)	angle shift (deg)	*S* (deg/RIU)
1	68.04	70.54	2.5	125
2	68.33	70.86	2.53	126.5
3	68.65	71.22	2.57	128.5
4	68.98	71.59	2.61	130.5
5	69.3	71.98	2.68	134
6	69.62	72.37	2.75	137.5
7	69.98	72.8	2.82	141
8	70.36	73.23	2.87	143.5
9	70.75	73.7	2.95	147.5
10	71.15	74.19	3.04	152

**Figure 5.**
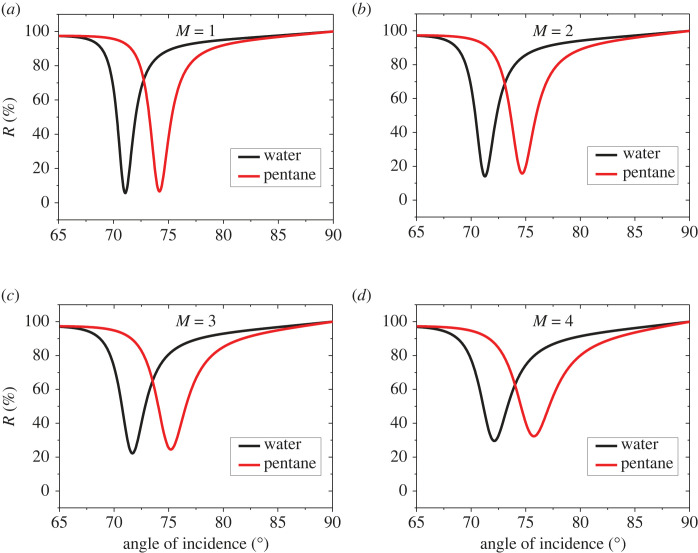
RECs of the device Prism/Ag/BaTiO_3_/Ag/G/SM at different numbers of graphene layers. (*a*) *M* = 1 (*b*) *M* = 2 (*c*) *M* = 3 and (*d*) *M* = 4.

**Figure 6.**
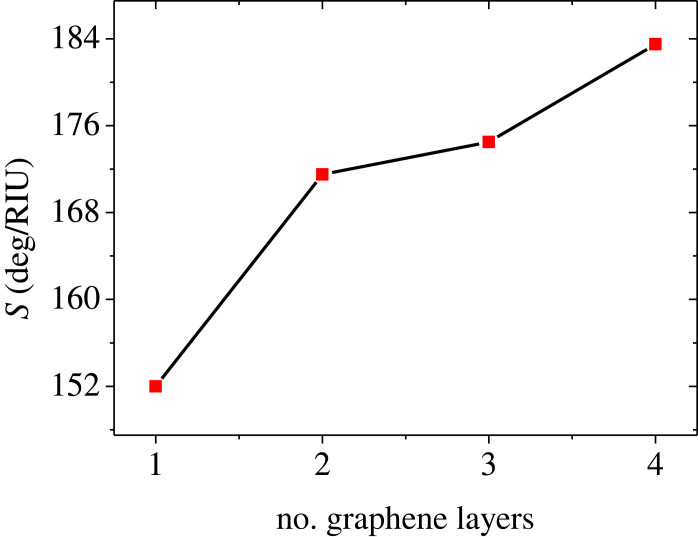
Sensitivity of the device Prism/Ag/BaTiO_3_/Ag/G/SM versus the number of graphene sheets at *d*_1_ = 45 nm, *d*_2_ = *P* × 1 nm, *P* = 10 and *d*_3_ = 8 nm.

To find the optimal number of graphene sheets which provides the highest sensitivity, we have studied the sensor performance for different numbers of graphene layers. The device now has the thicknesses: *d*_1_ = 45 nm, *d*_2_ = *P* × 1.0 nm where *P* = 10, and *d*_3_ = 8 nm. The reflectance spectra are plotted in figure 5 for numbers of graphene sheets of *M* = 1 nm (figure 5*a*), *M* = 2 (figure 5*b*), *M* = 3 (figure 5*c*) and *M* = 4 (figure 5*d*). It is observed that the rise in the number of graphene sheets leads to a dip shift towards higher REAs with an obvious increase in the minimum intensity of reflectivity as can be seen in figure 5. The calculations of dip positions, angular shift and sensitivity of the SPR sensor for different numbers of graphene layers (*M* = 1 to 4) are presented in table 3. It should be noted that, with the rise in the number of graphene layers, the sensitivity improves. The sensitivity has been found as 152, 171.5, 174.5 and 183.5 deg/RIU for *M* = 1, *M* = 2, *M* = 3 and *M* = 4, respectively. Figure 6 shows the variation of sensitivity for different numbers for graphene sheets. For *M* > 4, the resonant dip starts to disappear. Hence, *M* = 4 can be the best choice for the Prism/Ag/BaTiO_3_/Ag/G/SM device performance. It is worth mentioning that the reflectance dips disappear if the number of graphene layers exceeds *M* = 4.

**Table 3.** Variation of the sensitivity of the device Prism/Ag/BaTiO_3_/Ag/G/SM at different numbers of graphene sheets at *d*_1_ = 45 nm, *d*_3_ = 8 nm and *d*_2_ = P × 1 nm and P = 10.

number of G layers	position of resonant dip for water (deg)	position of resonant dip for pentane (deg)	angle shift (deg)	*S* (deg/RIU)
1	71.15	74.19	3.04	152
2	71.26	74.69	3.43	171.5
3	71.69	75.18	3.49	174.5
4	72.10	75.77	3.67	183.5

**Figure 7.**
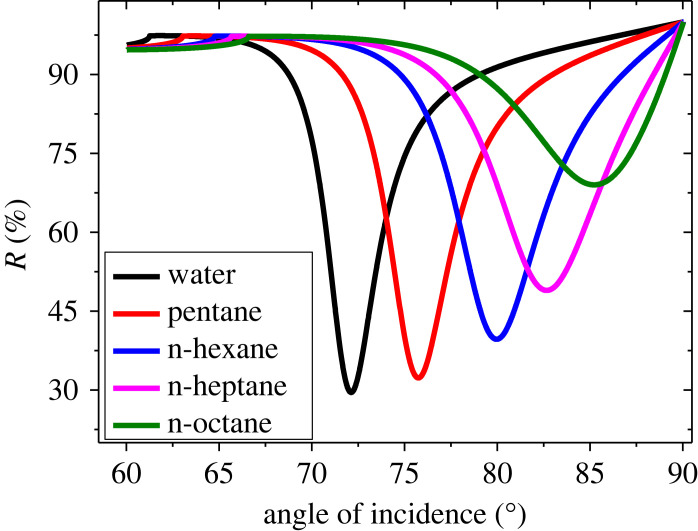
Reflectance curves of the device Prism/Ag/BaTiO_3_/Ag/G/SM at different organic compounds at *d*_1_ = 45 nm, *d*_3_ = 8 nm, *d*_2_ = P × 1.0 nm where *P* = 10 and *d*_4_ = *M* × 0.34 nm where *M* = 4.

**Figure 8.**
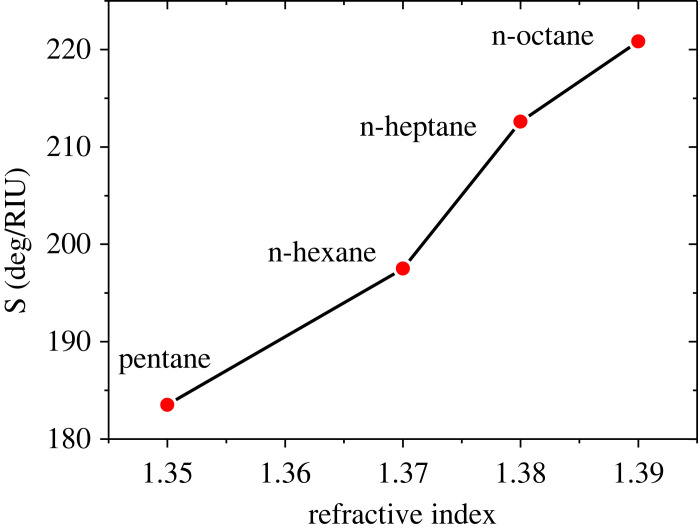
Sensitivity of the device Prism/Ag/BaTiO_3_/Ag/G/SM versus the RI of the OCDs at *d*_1_ = 45 nm, *d*_3_ = 8 nm, *d*_2_ = *P* × 1.0 nm where *P* = 10 and d_4_ = *M* × 0.34 nm where M = 4.

Based on the optimized values of BaTiO_3_ and G layer thicknesses, we now consider the response of the SPR sensor for various OCDs. Figure 7 shows the resonant dips of the reflectance when different analyte materials are considered. When the water is treated as an SM, the position of the resonant dip is found at 72.10 deg. It is observed that REA moves to a higher angle region as OCDs are treated as an SM. The dip angular positions are found at 75.77, 80, 82.73 and 85.35 deg and the sensitivity is also found as 183.5, 197.5, 212.6 and 220.83 deg/RIU for the OCDs of pentane, n-Hexane, n-Heptane and n-Octane, respectively. The calculations of dip positions, angular shift and sensitivity for various OCDs are presented in table 4. The sensitivity versus the RI of OCDs is displayed in figure 8 as the device Prism/Ag/BaTiO_3_/Ag/G/SM is characterized by the thicknesses of *d*_1_ = 45 nm, *d*_2_ = *P* × 1.0 nm where *P* = 10, *d*_3_ = 8 nm and *d*_4_ = *M* × 0.34 nm where *M* = 4. It is clear that the highest sensitivity of 220.83 deg/RIU can be obtained when n-Octane acts as an SM. The sensor shows good performance for the detection of OCDs with high sensitivity compared to most recent papers published in the field of chemical sensing as shown in table 5.

**Table 4.** Variation of the sensitivity of the device Prism/Ag/BaTiO_3_/Ag/G/SM for different OCDs at *d*_1_ = 45 nm, *d*_3_ = 8 nm, *d*_2_ = P × 1.0 nm where *P* = 10 and *d*_4_ = *M* × 0.34 nm where *M* = 4.

the compounds	RI	position of resonant dip (deg)	angle shift (deg)	*S* (deg/RIU)
water	1.33	72.10	-	-
pentane	1.35	75.77	3.67	183.5
n-hexane	1.37	80	7.9	197.5
n-heptane	1.38	82.73	10.63	212.6
n-octane	1.39	85.35	13.25	220.83

**Table 5.** Comparison between the current work and the most recent sensors.

assembly	year	S (deg/RIU)	reference
chemical sensor with low dark current Ge photodetector	2015	18.8	[32]
sensing of water concentration in ethanol solution using a photonic crystal	2022	144.369	[33]
conventional SPR sensor employing an additional plasma layer	2021	103	[34]
SPR sensor based on black phosphorus nanomaterial	2022	124	[35]
titanium dioxide coated etched fibre Bragg grating sensors	2020	11.9	[36]
SPR biosensor based on Ag-BaTiO_3_-Ag-graphene layers for the detection of organic compounds	2022	220.83	current work

## 4. Conclusion

Despite the fact that organic chemicals are essential for industry, some of them can be extremely toxic when released into the environment. Additionally, the chemical waste of organic chemicals can be a source of gaseous environmental pollution and substantial concern to human health. In this paper, we have proposed an SPR-based chemical sensor for detecting some organic chemicals. The proposed configuration employs two layers of Ag, BaTiO_3_ and graphene. Three structures have been examined: Prism/Ag/G/SM (assembly 1), Prism/Ag/BaTiO_3_/G/SM (assembly 2) and Prism/Ag/BaTiO_3_/Ag/G/SM (assembly 3). It is found that assembly 3 has the highest sensitivity of 125 deg/RIU, FoM of 102.45 1/RIU and a very low FWHM. The sensitivity has been investigated with the thickness of the BaTiO_3_ layer and the number of graphene sheets. The maximum sensitivity is attained at a BaTiO_3_ layer thickness of 10 nm and a number of graphene sheets of 4. The sensitivity of the assembly Prism/Ag/BaTiO_3_/Ag/G/SM has been investigated with a variety of organic compounds and the highest sensitivity of 220 deg/RIU has been reached for n-Octane analyte medium. Hence, it is expected that the proposed sensor is a strong candidate for various applications in biomedical, industrial and chemical detection.

## Data Availability

A Mathcad program that shows all calculations and figures are provided in the electronic supplementary material [37].

## References

[1] Heideman RG, Kooyman RPH, Greve J. 1993 Performance of a highly sensitive optical waveguide MachZehnder interferometer immunosensor. Sens. Actuators B Chem. **10**, 209-217. (10.1016/0925-4005(93)87008-D)

[2] El-Agez TM, Taya SA. 2011 An extensive theoretical analysis of the 1 : 2 ratio rotating polarizer–analyzer Fourier ellipsometer. Phys. Scr. **83**, 025701. (10.1088/0031-8949/83/02/025701)

[3] Rowe-Taitt CA, Hazzard JW, Hoffman KE, Cras JJ, Golden JP, Ligler FS. 2000 Simultaneous detection of six biohazardous agents using a planar waveguide array biosensor. Biosens. Bioelectron. **15**, 579-589. (10.1016/S0956-5663(00)00122-6) 11213218

[4] Singh Y, Paswan MK, Raghuwanshi SK. 2021 Sensitivity enhancement of SPR sensor with the black phosphorus and graphene with Bi-layer of gold for chemical sensing. Plasmonics **16**, 1781-1790. (10.1007/s11468-020-01315-3)

[5] Tamersit K, Djeffal F. 2016 Double-gate graphene nanoribbon field-effect transistor for DNA and gas sensing applications: simulation study and sensitivity analysis. IEEE Sens. J. **16**, 4180-4191. (10.1109/JSEN.2016.2550492)

[6] Pandey PS, Raghuwanshi SK, Singh Y. 2022 Enhancement of the sensitivity of a surface plasmon resonance sensor using a nobel structure based on barium titanate–graphene –silver. Opt. Quant. Electron. **54**, 417. (10.1007/s11082-022-03803-8)

[7] Paul S, Paul D, Fern GR, Ray AK. 2011 Surface plasmon resonance imaging detection of silver nanoparticle-tagged immunoglobulin. J. R. Soc. Interface **8**, 1204-1211. (10.1098/rsif.2010.0747) 21325318 PMC3119885

[8] Miyazaki CM, Shimizu FM, Mejía-Salazar JR, Oliveira ON, Ferreira M. 2017 Surface plasmon resonance biosensor for enzymatic detection of small analytes. Nanotechnology **28**, 145501. (10.1088/1361-6528/aa6284) 28287081

[9] Babaei E, Sharifi Z, Gordon R. 2019 Improving sensitivity of existing surface plasmon resonance systems with grating-coupled short-range surface plasmons. J. Opt. Soc. Am. B **36**, F144-F148. (10.1364/JOSAB.36.00F144)

[10] Srivastava A, Verma A, Prajapati YK. 2021 Theoretical study of hazardous carbon- di-oxide gas sensing using MIM structure-based SPR sensing scheme. IET Optoelectron. **15**, 167-177. (10.1049/ote2.12035)

[11] Varasteanu P. 2020 Transition metal dichalcogenides/gold-based surface plasmon resonance sensors: exploring the geometrical and material parameters. Plasmonics **15**, 243-253. (10.1007/s11468-019-01033-5)

[12] El-Amassi DM, Taya SA. 2017 Reflection through a parallel-plate waveguide formed by two graphene sheets. Photonics Nanostructures - Fundam. Appl. **24**, 53-57. (10.1016/j.photonics.2017.03.008)

[13] Gerber JA, Berweger S, O'Callahan BT, Raschke MB. 2014 Phase-resolved surface plasmon interferometry of graphene. Phys. Rev. Lett. **113**, 055502. (10.1103/PhysRevLett.113.055502) 25126927

[14] Wu F, Liu D, Xiao S. 2021 Bandwidth-tunable near-infrared perfect absorption of graphene in a compound grating waveguide structure supporting quasi-bound states in the continuum. Opt. Express **29**, 41 975-41 989. (10.1364/OE.446270)

[15] Wu F, Chen M, Chen Z, Yin C. 2021 Omnidirectional terahertz photonic band gap broaden effect in one-dimensional photonic crystal containing few-layer graphene. Opt. Commun. **490**, 126898. (10.1016/j.optcom.2021.126898)

[16] Karki B, Sharma S, Singh Y, Pal A. 2021 Sensitivity enhancement of surface plasmon resonance biosensor with 2-D Franckeite nanosheets. Plasmonics **17**, 71-78. (10.1007/s11468-021-01495-6) PMC8763424 35069047

[17] Han F, Yu B, Meng F, Zhao C, Zhou L. 2021 Tunable mid-infrared optical properties of monolayer black phosphorus through au nanotriangle arrays via electric field reflection and surface plasmon polaritons. Plasmonics **16**, 1729-1734. (10.1007/s11468-021-01426-5)

[18] Sathya N, Karki B, Rane KP, Jha A, Pal A. 2022 Tuning and sensitivity improvement of Bi-metallic structure-based surface plasmon resonance biosensor with 2-D ξ-tin selenide nanosheets. Plasmonics **17**, 1001-1008. (10.1007/s11468-021-01565-9) 35069047 PMC8763424

[19] Simsek E. 2013 Improving tuning range and sensitivity of localized SPR sensors with graphene. IEEE Photon Technol. Lett. **25**, 867-870. (10.1109/LPT.2013.2253316)

[20] Xie J et al. 2019 "Optical properties of chemical vapor deposition-grown PtSe2 characterized by spectroscopic ellipsometry. 2D Mater. **6**, 035011. (10.1088/2053-1583/ab1490)

[21] Uniyal A, Chauhan B, Pal A, Srivastava V. 2022 InP and graphene employed surface plasmon resonance sensor for measurement of sucrose concentration: a numerical approach. Opt. Eng. **61**, 057103. (10.1117/1.OE.61.5.057103)

[22] Karki B, Trabelsi Y, Uniyal A, Pal A. 2022 Zinc sulfide, silicon dioxide, and black phosphorus based ultra-sensitive surface plasmon biosensor. Opt. Quant. Electron **54**, 107. (10.1007/s11082-021-03480-z)

[23] Karki B, Uniyal A, Pal A, Srivastava V. 2022 Advances in surface plasmon resonance-based biosensor technologies for cancer cell detection. Int. J. Optics **2022**, Article ID 1476254, 10 pages. (10.1155/2022/1476254)

[24] Uniyal A, Srivastava G, Pal A, Taya S, Muduli A. 2023 Recent advances in optical biosensors for sensing applications: a review. Plasmonics **18**, 735-750. (10.1007/s11468-023-01803-2)

[25] Karki B, Jha A, Pal A, Srivastava V. 2022 Sensitivity enhancement of refractive index-based surface plasmon resonance sensor for glucose detection. Opt. Quant. Electron **54**, 595. (10.1007/s11082-022-04004-z)

[26] You JW, Threlfall E, Gallagher DFG, Panoiu NC. 2018 Computational analysis of dispersive and nonlinear 2D materials by using a GS-FDTD method. J. Opt. Soc. Am. B **35**, 2754-2763. (10.1364/JOSAB.35.002754)

[27] Karvounis A, Timpu F, Vogler-Neuling VV, Savo R, Grange R. 2020 Barium titanate nanostructures and thin films for photonics. Adv. Opt. Mater. **8**, 2001249. (10.1002/adom.202001249)

[28] Aly AH, Awasthi SK, Mohamed D, Matar ZS, Al-Dossari M, Amin AF. 2021 Study on a one-dimensional defective photonic crystal suitable for organic compound sensing applications. RSC Adv. **11**, 32973. (10.1039/D1RA06513K) 35493603 PMC9042218

[29] Lin Z, Jiang L, Wu L, Guo J, Dai X, Xiang Y, Fan D. 2016 Tuning and sensitivity enhancement of surface plasmon resonance biosensor with graphene covered Au MoS2-Au films. IEEE Photon J. **8**, 1-8.

[30] Liu N, Wang S, Cheng Q, Pang B, Lv J. 2021 High Sensitivity in Ni-Based SPR Sensor of Blue Phosphorene/Transition Metal Dichalcogenides Hybrid Nanostructure. Plasmonics **16**, 1567-1576. (10.1007/s11468-021-01421-w)

[31] Bruna M, Borini S. 2009 Optical constants of graphene layers in the visible range. Appl. Phys. Lett. **94**, 03190. (10.1063/1.3073717)

[32] Zang K et al. 2015 Microring bio-chemical sensor with integrated low dark current Ge photodetector. Appl. Phys. Lett. **106**, 101111. (10.1063/1.4915094)

[33] Taya SA, Sharma A, Doghmosh N, Colak I. 2022 Detection of water concentration in ethanol solution using a ternary photonic crystal-based sensor. Mater. Chem. Phys. **279**, 125772. (10.1016/j.matchemphys.2022.125772)

[34] Taya SA, Al-Ashi NE, Ramahi OM, Colak I, Amiri IS. 2021 Surface plasmon resonance-based optical sensor using a thin layer of plasma. J. Opt. Soc. Am. B **38**, 2362-2367. (10.1364/JOSAB.420129)

[35] Almawgani AHM, Daher MG, Taya SA, Olaimat MM, Alhawari ARH, Colak I. 2022 Detection of blood plasma concentration theoretically using SPR-based biosensor employing black phosphor layers and different metals. Plasmonics **17**, 1751-1764. (10.1007/s11468-022-01662-3)

[36] Singh Y, Sadhu A, Raghuwanshi SK. 2020 Development and experimental analysis of titanium dioxide (TiO2) coated etched fiber bragg grating sensor for chemical sensing. IEEE Sens. J. **20**, 8528. (10.1109/JSEN.2020.2983263)

[37] Taya SA, Daher MG, Almawgani AHM, Hindi AT, Colak I. 2023 A surface plasmon resonance nanostructure containing graphene and BaTiO_3_ layers for sensitive defection of organic compounds. Figshare. (10.6084/m9.figshare.c.6697377) PMC10282577 37351487

